# Oral Microbiome Stamp in Alzheimer’s Disease

**DOI:** 10.3390/pathogens13030195

**Published:** 2024-02-23

**Authors:** Argul Issilbayeva, Aiym Kaiyrlykyzy, Elizaveta Vinogradova, Zharkyn Jarmukhanov, Samat Kozhakhmetov, Aliya Kassenova, Madiyar Nurgaziyev, Nurislam Mukhanbetzhanov, Dinara Alzhanova, Gulnaz Zholdasbekova, Sholpan Askarova, Almagul R. Kushugulova

**Affiliations:** 1 Center for Life Sciences, National Laboratory Astana, Nazarbayev University, Astana Z05H0P9, Kazakhstan; argul.issilbayeva@nu.edu.kz (A.I.); aiym.kaiyrlykyzy@nu.edu.kz (A.K.); st.paulmississippi@gmail.com (E.V.); zharkyn.jarmukhanov@nu.edu.kz (Z.J.); skozhakhmetov@nu.edu.kz (S.K.); aliya.kassenova@nu.edu.kz (A.K.); madiyar.nurgaziyev@nu.edu.kz (M.N.); nurislam.mukhanbetzhanov@nu.edu.kz (N.M.); gulnazzholdasbekova@gmail.com (G.Z.); 2 Department of Neurology, Medical University Astana, Astana Z05H0P9, Kazakhstan; dinara.alzhanowa@yandex.ru; 3 Medical University Karaganda, Karaganda M01K3B6, Kazakhstan; 4 JSC “National Research Cardiac Surgery Center”, Astana 010000, Kazakhstan

**Keywords:** oral cavity, microbiome, 16S rRNA, sequencing, metabolic pathways, Alzheimer’s disease, Kazakhstan population

## Abstract

Recent studies have suggested that periodontal disease and alterations in the oral microbiome may be associated with cognitive decline and Alzheimer’s disease (AD) development. Here, we report a case-control study of oral microbiota diversity in AD patients compared to healthy seniors from Central Asia. We have characterized the bacterial taxonomic composition of the oral microbiome from AD patients (n = 64) compared to the healthy group (n = 71) using 16S ribosomal RNA sequencing. According to our results, the oral microbiome of AD has a higher microbial diversity, with an increase in *Firmicutes* and a decrease in *Bacteroidetes* in the AD group. LEfSe analysis showed specific differences at the genus level in both study groups. A region-based analysis of the oral microbiome compartment in AD was also performed, and specific differences were identified, along with the absence of differences in bacterial richness and on the functional side. Noteworthy findings demonstrated the decrease in periodontitis-associated bacteria in the AD group. Distinct differences were revealed in the distribution of metabolic pathways between the two study groups. Our study confirms that the oral microbiome is altered in AD. However, a comprehensive picture of the complete composition of the oral microbiome in patients with AD requires further investigation.

## 1. Introduction

Alzheimer’s disease (AD) is a gradually worsening neurodegenerative condition marked by memory decline, significant alterations in personality and conduct, and an inability to perform routine daily tasks in its advanced stages. The likelihood of developing AD rises with age, impacting around 10% of individuals between 65 and 75 years old and affecting 32% of those aged 80 and older [1,2]. According to the World Health Organization (WHO), the incidence of AD is increasing every year. Thus, it is postulated that there could be a threefold increase in the number of AD patients by 2050, and the most significant increases in dementia will happen in low- and middle-income countries [3]. In Kazakhstan, as well as in other countries of the world, over the past decades, there has been an increase in the number of older adults. However, the Kazakh population is poorly represented in global studies of dementia.

Numerous factors that elevate the risk of Alzheimer’s disease (AD) have been identified. These include both non-modifiable (age, gender, family history, genetic) and modifiable factors such as low education, midlife hypertension, high cholesterol, physical inactivity, obesity and diabetes, and many others. One of the critical factors that has been influencing human health and increasingly attracting the attention of scientists during the last two decades is the human microbiota.

The human microbiota is referred to as the genomic content of microorganisms colonizing various anatomical locations like the digestive system, respiratory system, urogenital system, etc., where they form multifaceted and distinct ecosystems that adapt to the environmental conditions and closely interact with the host organism [1,2]. These interactions have a tremendous impact on human physiology in healthy individuals and during an illness. The gut harbors the most extraordinary microbial diversity and load, which play a crucial role in influencing both human health and the development of various diseases. Recent research strongly indicates that the microbiota in the gastrointestinal tract can influence brain functions and contribute to the development of AD [4,5,6,7]. The oral microbiome, ranking second in the microbiomes of the human body, also plays a crucial role in both oral and systemic diseases and can influence brain functions [8,9] through its potential to provoke neuroinflammation [10]. Studies have shown that the oral microbiome can impact the pathophysiological and immunoinflammatory mechanisms of Alzheimer’s disease [11]. Factors such as aging, local inflammation, and systemic diseases can contribute to microbial dysbiosis, potentially leading to the development or exacerbation of Alzheimer’s disease [12]. Prebiotic compounds and probiotic strains are a potential therapeutic approach for the modulation of the oral microbiome [11].

Many studies have provided evidence of a connection between periodontal disease and both neurodegeneration and cognitive decline [13,14,15]. Chang et al. reported that chronic periodontitis of 10 years was associated with a 1707-fold increased risk of developing AD [16]. A nationwide retrospective cohort study carried out in Taiwan found that individuals with chronic periodontitis and gingivitis had a higher likelihood of developing dementia when compared to those with healthy gum conditions [17]. Moreover, recent evidence has demonstrated a direct causal relationship between the oral microbiome and AD [18,19,20,21,22]. For example, *P. gingivalis*, the most common bacterium associated with periodontal disease, was able to cause the buildup of beta-amyloid plaques and neurofibrillary tangles after an experimental oral infection in mice [23]. In turn, the content of serum antibodies to *P. gingivalis* was increased in patients with AD [24], and the enzyme gingipain produced by *P. gingivalis* was found in the brains of patients with Alzheimer’s disease [25]. Dominy et al. demonstrated that oral administration of small-molecule gingipain inhibitors blocked gingipain-induced neurodegeneration, reduced *P. gingivalis* levels, and reduced Aβ42 production in mouse neural tissue resulting from *P. gingivalis* brain infection [23]. Chronic systemic *P. gingivalis* infection has also been found to induce Aβ accumulation in inflammatory monocytes/macrophages via activation of CatB/NF-κB signaling [26]. Recent studies suggest that *Aggregatibacter actinomycetemcomitans* has been implicated in Alzheimer’s disease through its potential to induce inflammatory responses and affect the central nervous system [27,28]. In vitro studies have shown that *A. actinomycetemcomitans* can upregulate inflammatory cytokines and toll-like receptors in brain cells, leading to increased expression of pro-inflammatory cytokines such as IL-1β, IL-6, and TNF-α [29]. Additionally, *A. actinomycetemcomitans* has been found to induce the secretion of β-amyloid, a hallmark protein associated with Alzheimer’s disease [27]. Furthermore, extracellular RNA in outer-membrane vesicles of A. actinomycetemcomitans has been shown to cross the blood–brain barrier and induce inflammation [30].

In addition, evidence indicates significant differences in the quantity and quality of the oral microbiome in patients with AD compared to cognitively healthy age-matched individuals. For example, Liu et al. demonstrated lower abundance and biodiversity of the salivary microbiome in patients with Alzheimer’s disease than in healthy controls [19]. The authors note relatively high levels of *Moraxella*, *Leptotrichia*, and *Sphaerochaeta* and a significant decrease in the number of *Rothia* in the saliva of patients with AD [19]. However, these authors highlight the study’s limitations due to the absence of many periodontal bacteria in saliva that typically exist in the subgingival niche or calculus [31]. Thus, a complete picture of the composition of the oral microbiome and its relationship with AD requires further research. Thus, in the present study, we have investigated the diversity and composition of oral microbiomes isolated from local patients diagnosed with AD in comparison with healthy seniors and probed possible links between the oral microbiota and some clinical parameters.

## 2. Materials and Methods

### 2.1. Responders Recruitment

Participants were recruited from Astana and Almaty through partnerships with neurologists at healthcare facilities, including City Hospital No. 1 in Astana and Shashkin Clinic in Almaty, between September 2020 and April 2022.

The study employed a predefined algorithm and a case-control study design, focusing on older individuals with Alzheimer’s disease (cases) and cognitively healthy elderly individuals (controls). Alzheimer’s disease diagnosis adhered to the NINCDS-ADRDA criteria. Inclusion criteria for the “case” group comprised the following: (1) Alzheimer’s disease diagnosis; (2) age 50 or older at the time of diagnosis and data collection; (3) voluntary consent to participate and provide blood and oral swab samples. Inclusion criteria for the “control” group included the following: (1) absence of cognitive impairment, including memory deficits; (2) appropriate age and gender; (3) voluntary consent to participate and provide blood and stool samples. Exclusion criteria encompassed (1) severe somatic diseases and (2) unrelated mental disorders for the “case” group. The control group consisted of age-matched individuals without Alzheimer’s disease, selected to parallel the patient cohort in demographic characteristics.

**Figure 1:**
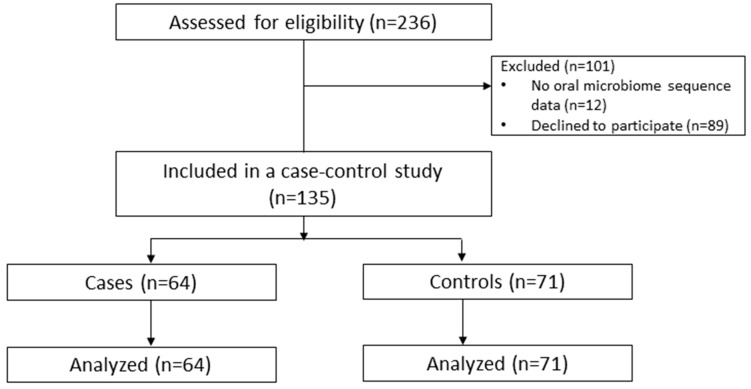
Diagram of the study design.

All participants (or their guardians) received comprehensive information from the coordinator regarding the research project’s objectives and provided written informed consent to participate. All patients underwent laboratory examination, with blood samples collected in a fasting state. Comorbidity data from participants, including those with dementia, were collected via self-report, caregiver reports, and medical records. The design of the study is provided in Figure 1.

### 2.2. Sample Collection and DNA Isolation

Before obtaining samples, participants were instructed to abstain from oral hygiene procedures for the preceding 12 h and to refrain from eating or drinking in the 2 h leading up to the study visit. Samples collection was performed according to the Human Microbiome Project, Core Microbiome Sampling Protocol [32], from various areas within the oral cavity, encompassing saliva, soft tissues, and hard tissues. The collection of saliva involved using a calibrated pipette to obtain samples from the floor of the mouth. Soft tissue specimens were acquired from multiple locations, including the dorsum of the tongue, hard palate, buccal mucosa, keratinized (attached) gingiva, palatine tonsils, and throat, using a DNA/RNA Shield Collection Tube w/ Swab (Zymo Research, Orange, CA, USA, R1107). Both supragingival plaque and subgingival plaque were collected from a minimum of four molar teeth, employing a Gracey curette. Equal numbers of plaque samples were included from two studies. DNA extraction was performed using the ZymoBIOMICS DNA Miniprep Kit (Zymo Research, Orange, CA, USA, D4300). A qualitative control of isolated DNA was conducted by electrophoresis using 1% agarose gel. The concentration and clarity of the isolated DNA samples were defined using an Invitrogen Qubit 3.0 Fluorometer (Invitrogen, Carlsbad, CA, USA); as a negative control, sterile water was served.

### 2.3. Periodontal Status Evaluation of Responders

The participants underwent a clinical examination conducted by a periodontologist, which involved a comprehensive gathering of complaints and medical histories, an assessment of the oral cavity, and an index evaluation of the periodontal tissue condition. The examination addressed patient-reported issues such as bleeding gums; the prescription and the circumstances of its occurrence; the frequency of abscess formation; the presence of bad breath; tooth mobility, as well as cosmetic concerns related to the movement of the frontal group of teeth; heightened tooth sensitivity to various stimuli; and disruptions in the function of the dental system. Parameters such as bone loss (%), bleeding on probing (BoP), periodontal probing depths (PPD), clinical loss of attachment (CAL), gingival recession (GR), and number of remaining teeth were indicated. Periodontal health was defined for areas with a probing depth (PPD) of ≤3 mm and an absence of bleeding during probing. The presence of periodontitis was determined in cases where probing depth (PPD) was ≥4 mm, clinical loss of attachment (CAL) was ≥2 mm, and radiographic bone loss accounted for >15%.

### 2.4. Statistical Analysis

#### 2.4.1. Clinical and Demographic Data Analysis

Statistical analysis was performed using Python 3.9.16. The normality was assessed by the Shapiro–Wilk test. The equality of variances was checked by Levene’s test. Quantitative data were compared using unpaired *t*-tests or Mann–Whitney U tests, as appropriate. Discrete variables comparison analysis was carried out by chi-squared tests or Fisher’s exact tests. Statistically significant results were determined in the case of *p* values < 0.05.

#### 2.4.2. Sequencing Data Analysis

The analysis method of 16S rRNA sequencing was performed on the Illumina NovaSeq 6000 platform in the Novogene laboratory (Beijing, China) following the standard Illumina protocols. The V3–V4 hypervariable regions of the 16S rRNA were employed for analysis.

For 16S amplicon sequencing data processing, the LotuS2 pipeline (Less OTU Scripts 2) was used. All reads were demultiplexed, quality filtered, and dereplicated by using a demultiplexer. Chimeric sequences were eliminated by employing UCHIME algorithms. Taxonomic postprocessing of amplicon sequences in LCA with sequence clustering UPARSE was performed using the SILVA database.

Statistical analysis and visualization of microbiome composition were performed in Python 3.9.16. The OTU table was rarefied to account for inter-sample variation; only OTUs present in at least 25% of the samples in either group were retained. All two-group comparisons were performed using the Mann–Whitney U test with the “SciPy 1.10.1” library, and correlations were calculated using Spearman’s ρ. No adjustment for multiple comparisons was applied when comparing correlations between significantly differentially abundant parameters; in all other cases, FDR-BH correction was applied. Alpha diversity was assessed using Shannon and Chao1 indices and using Faith phylogenetic diversity and the number of OTUs observed after filtration; beta diversity was assessed based on the unweighted UniFrac (U-UniFrac) distance using ANOSIM and PERMANOVA tests with 9999 permutations using the “scikit-bio 0.5.6” library. Ordination of between-sample composition was performed using principal coordinate analysis (PCoA). Differential analysis of taxonomic features was performed using “LEfSe” with a significance cutoff of *p* ≤ 0.05 and LDA > 2. Differential analysis of functional features was based on differences in medians with 95% CI estimation using the Hodges–Leghman estimator. Only differences in functional features with non-overlapping CIs were considered significant. Feature importance analysis was performed using a combination of gradient boosting decision trees (GB) and binomial logistic regression (LR) with leave-one-out cross-validation using the “scikit-learn 1.2.2” and “LightGBM 3.3.5” libraries without prior feature selection or hyperparameter tuning. ROC-AUC analysis was performed based on the leave-one-out predictions. Visualization was performed using the “Matplotlib 3.7.1”, “seaborn 0.11.2”, and “Sankey flow 0.3.8” libraries. Cladograms and L2FC bar plots were built using the LEfSe 1.0.8 visual tools.

#### 2.4.3. Metabolic Pathways Analysis

PICRUSt2 (phylogenetic investigation of communities by reconstruction of unobserved states) v2.5.0 with default parameters was used for the prediction of functional metagenomic data based on the 16S rRNA sequencing results [33]. In order to estimate the gene family copy numbers for each OTU, a reference tree with an NSTI (nearest sequenced taxon index) cutoff of 2 was employed. The MetaCyc Metabolic Pathway database was used to forecast the abundance of bacterial metabolic pathways.

## 3. Results

### 3.1. Responders Characteristics

The study overall enrolled 135 participants, 64 patients with Alzheimer’s disease (AD) and 71 healthy individuals (as shown in Figure 1). The average age of AD patients was 68 years; in the control group, it was 67 years. A total of 34 AD patients and 45 controls were recruited in Almaty, and 30 AD patients and 26 controls were recruited from Astana.

The control group of individuals matched for age, sex, and ethnicity to the AD patients (Table 1). There were no significant differences in periodontitis frequency between the two studied groups, *p* > 0.05. Statistically significant differences were obtained in laboratory parameters; total cholesterol (TC), low density lipoprotein (LDL), triglyceride (TG), alanine transaminase (ALT), and aspartate transaminase (AST) were significantly lower in AD patients compared to the control group (Table 1).

There were no significant differences in periodontitis frequency between the two studied groups or between female and male subjects, *p* > 0.05. Periodontal parameters (such as bone loss (%), BoP, PPD, CAL, GR, and number of remaining teeth) were similarly distributed between the groups, *p* > 0.05. More details on the distribution of periodontal parameters can be found in the Supplementary Material (Tables S1 and S2).

For further analysis, the main study group was divided into subgroups depending on regions: Almaty-AD and Astana-AD, respectively. The clinical and demographic characteristics are indicated in Table 2. The age of the two subgroups demonstrated statistical differences; the Almaty-AD patients were younger than the Astana-AD patients, *p* = 0.002. BMI and MMSE were significantly higher in the Almaty-AD subgroup, *p* = 0.03 and *p* = 0.0002, respectively. PFHDEM (family history of dementia) predominated in the Astana-AD subgroup.

### 3.2. Oral Microbiome Compartment in AD and Control Groups

After filtering 8570 OTUs (29,261,786 reads) remaining in the matrix, the total reads in the matrix were 29,261,786. Reads processed: 32,974,292. Accepted (high qual): 25,687,589 (110,060 end-trimmed). Rejected: 7,286,703, with an average sequencing depth of 216,753.97 reads per sample.

**Figure 2:**
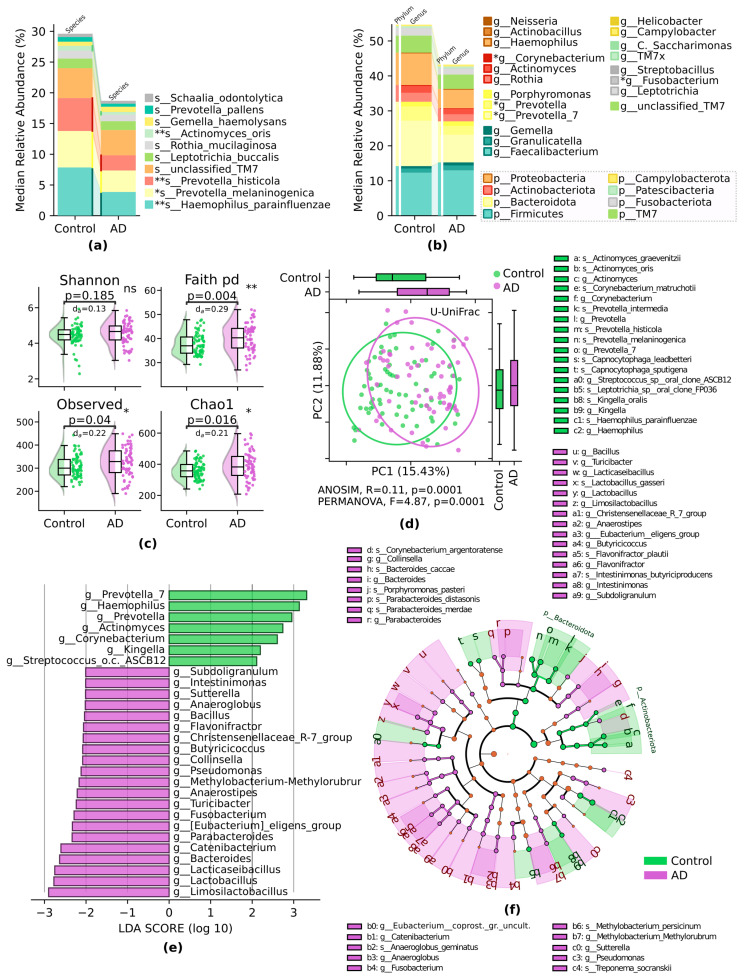
Oral microbiome compositional dissimilarities in AD and control groups: (**a**) median relative abundance of most abundant species; (**b**) median relative abundance of the top genera of most abundant phyla; Mann–Whitney U test; (**c**) α-diversity. Shannon and Chao1 indices. Number of observed OTUs and Faith phylogenetic diversity (pd). Mann–Whitney U test, with abs. Cliff’s delta effect size (normalized U statistic) ^a^; (**d**) β-diversity. Principal coordinate analysis (PCoA) ordination based on the unweighted UniFrac (U-UniFrac) distance. ANOSIM R, PERMANOVA F with permutations; (**e**) LefSe bar plot, LDA < 2 at the genus level; (**f**) LefSe cladogram at the genus and species levels. * *p* ≤ 0.05, ** *p* ≤ 0.01. ^a^ Cliff’s delta quantifies how often values in one distribution are higher than in the second distribution.

According to our results, the relative abundance of microbial taxa at the phylum level demonstrated specific differences; Firmicutes increased in the AD group. However, this difference did not show any statistical significance, while there was a decrease in *Bacteroidota* in the AD group at the tendency level, *p* = 0.06 (Figure 2a,b). Statistically significant differences between the two study groups were obtained at the species level; four species were decreased in AD compared to the control group: *s_Haemophilus_parainfluenzae*, *p* = 0.009; *s_Prevotella_melaninogenica*, *p* = 0.02; *s_Prevotella_histicola*, *p* = 0.01; *s__Actinomyces_oris*, *p* = 0.004 (Figure 2a). The α-diversity differed between study groups by Faith PD (*p* = 0.004), observed (*p* = 0.04), and Chao1 (*p* = 0.016) indexes (Figure 2c) and was higher in the AD group compared to the control. β-diversity also showed significant differences between the two groups by unweighted UniFrac: PERMANOVA, *p* = 0.0001; ANOISM, *p* = 0.0001, respectively (Figure 2d) and was higher in the AD group. LefSe at the LDA > 2 levels showed enrichment of specific taxa in the AD group, and beneficial bacteria were from *Limosilactobacillus*, *Lactobacillus*, *Lacticaseibacillus*, *Bacteroides*, *Catenibacterium*, *Parabacteroides*, *Eubacterium_eligens_group*, *Fusobacterium*, *Turicibacter*, and *Anaerostipes genera* (Figure 2e), which are described in more detail on the cladogram (Figure 2f).

### 3.3. Oral Microbiome Compartment in Astana-AD and Almaty-AD Subgroups

**Figure 3:**
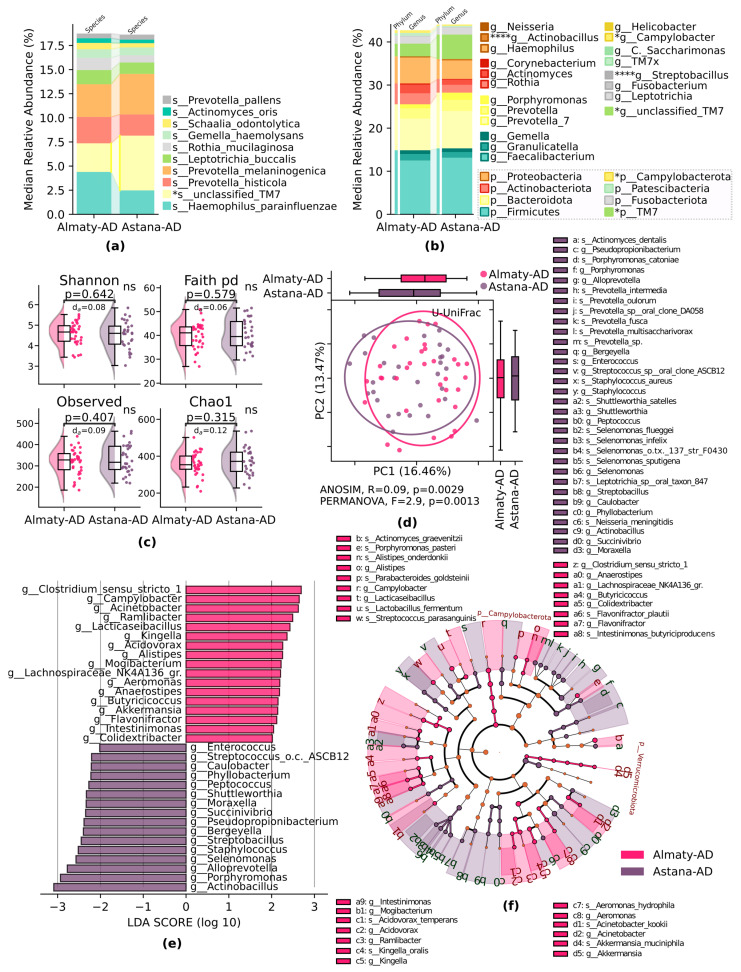
Oral microbiome compositional dissimilarities in AD subgroups in Almaty and Astana regions: (**a**) median relative abundance of most abundant species; (**b**) median relative abundance of the top genera of most abundant phyla; Mann–Whitney U test; (**c**) α-diversity. Shannon and Chao1 indices. Number of observed OTUs and Faith phylogenetic diversity (pd). Mann–Whitney U test, with abs. Cliff’s delta (d_a_) effect size (normalized U statistic); (**d**) β-diversity. Principal coordinate analysis (PCoA) ordination based on the unweighted UniFrac (U-UniFrac) distance. ANOSIM R, PERMANOVA F with permutations; (**e**) LefSe bar plot, LDA < 2 at the genus level; (**f**) LefSe cladogram at the genus and species levels. * *p* ≤ 0.05, **** *p* ≤ 0.0001.

Analysis of taxonomy in AD between the Almaty and Astana regions demonstrated the following significant differences: at the species level, *s_unclassified_TM7*, *p* < 0.05, prevailed in the Astana-AD subgroup (Figure 3a); at the genus level, *g_Actinobacillus*, *p* < 0.0001, and *g_unclassified_TM7*, *p* < 0.05, prevailed in the Astana-AD subgroup, while *g_Streptobacillus*, *p* < 0.0001, and *g_Campylobacter*, *p* < 0.05, prevailed in the Almaty-AD subgroup (Figure 3b). α-diversity did not show any differences by all indexes, *p* > 0.05 (Figure 3c). At the same time, unweighted UniFrac analysis of β-diversity demonstrated significant compositional differences, PERMANOVA *p* = 0.001, ANOISM *p* = 0.002, higher in the Astana-AD subgroup (Figure 3d). LefSe analysis of taxa revealed specific alterations in AD between two regions, *g_Clostridium_sensu_stricto1*, *g_Campylobacter*, *g_Acinetobacter*, *g_Ramlibacter*, *g_ Lacticaseibacillus*, *g_Kingella* were the most abundant taxa in the Astana-AD; *g_Actinobacillus*, *g_Porphyromonasadaceae*, *g_Alloprevotella*, *g_Selenomonas*, *g_Staphylococcus*, *g_Streptobacillus*, *g_Bergeyella* were the most abundant taxa in the Almaty-AD group (Figure 3e), which are introduced in greater detail in the cladogram (Figure 3f).

### 3.4. Correlation Interactions between AD and Control Group

**Figure 4:**
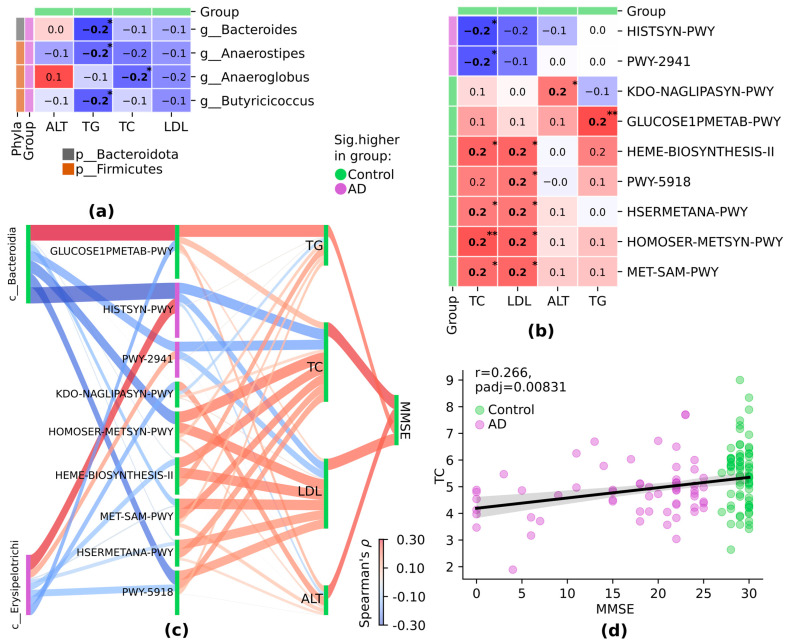
Significant correlations between differentially abundant features in AD and control groups: (**a**) between taxa and clinical parameters; (**b**) between pathways and clinical parameters; (**c**) between taxa, pathways, clinical parameters, and MMSE. Sankey plot of significant correlations. (**d**) Scatter plot comparing TC levels and MMSE scores. Spearman’s ρ, *p* ≤ 0.05; TC = total cholesterol; LDL = low-density lipoprotein; ALT = alanine transaminase; TG = triglycerides; MMSE = Minimized Mental State Examination. For differentially abundant features, a group with a higher relative abundance is highlighted: in green, the relative abundance is higher in the control group, and in purple, it is higher in the AD group. * *p* ≤ 0.05, ** *p* ≤ 0.01.

According to correlation analysis, specific correlations between taxa and clinical parameters at the species level were observed: *s__Anaeroglobus_geminatus* negatively correlated with cholesterol level, *s__Corynebacterium_argentoratense* negatively correlated with TG level, and positive correlations were observed between *s__Haemophilus_parainfluenzae* and cholesterol and *s__Leptotrichia_sp._oral_clone_FP036* and ALT level (Figure 4a).

Correlation analysis of taxa and metabolic pathways demonstrated several correlations; *s_Flavonifactor_plautii*, *s_Lactobaccillus_gassery*, *s_Intestinomionas_butyriciproducens*, *s_Treponema_socranskii*, and Porphyromonas_pasteri demonstrated the majority of correlation networks, precisely positive in the AD group with a large scale of pathways (Figure S1). *s_Haemofillus_parainfluenzae*, *s_Prevotella_melaninogenica*, *s_Prevotella_histicolla*, and *s_Prevotella_intermedia* displayed positive correlations with the large scale of pathways in the control group (Figure S1).

In correlation analysis of pathways and clinical parameters, HISTSYN-PWY and PWY-2941 pathways demonstrated a negative correlation with cholesterol level; HEME-BIOSYNTHESIS-II, HSERMETANA-PWY, HOMOSER-METSYN-PWY, and MET-SAM-PWY positively correlated with cholesterol and LDL levels; PWY-5918 showed a positive correlation with LDL level only; and GLUCOSE1PMETAB-PWY positively correlated with TG (Figure 4b).

Sankey plot analysis demonstrated specific connections between two key taxa, *c__Bateroidia* and *c__Erysipelotrichi,* nine metabolic pathways, four clinical parameters, and MMSE (Figure 4c). Notably, the two taxa demonstrated opposite correlation (*c__Bacteroidia* negative, *c__Erysipelotrichi* positive) regarding HISTSYN-PWY and PWY-2941, associated with AD, which in turn negatively correlated with cholesterol and LDL, and the latter positively correlated with MMSE (Figure 4c). PWY-5918 was associated with the control group and negatively correlated with both taxa; meanwhile, it positively correlated with TC, LDL levels, and MMSE (Figure 4c,d).

The distribution of metabolic pathways in AD and control groups is provided in Figure S2. A total of 15 pathways demonstrated statistical significance in the AD group and, 22 pathways did so in the control group. All correlations between significantly abundant taxa and significantly abundant pathways are provided in Table S3.

### 3.5. Correlation Interactions between Astana-AD and Almaty-AD Subgroups

**Figure 5:**
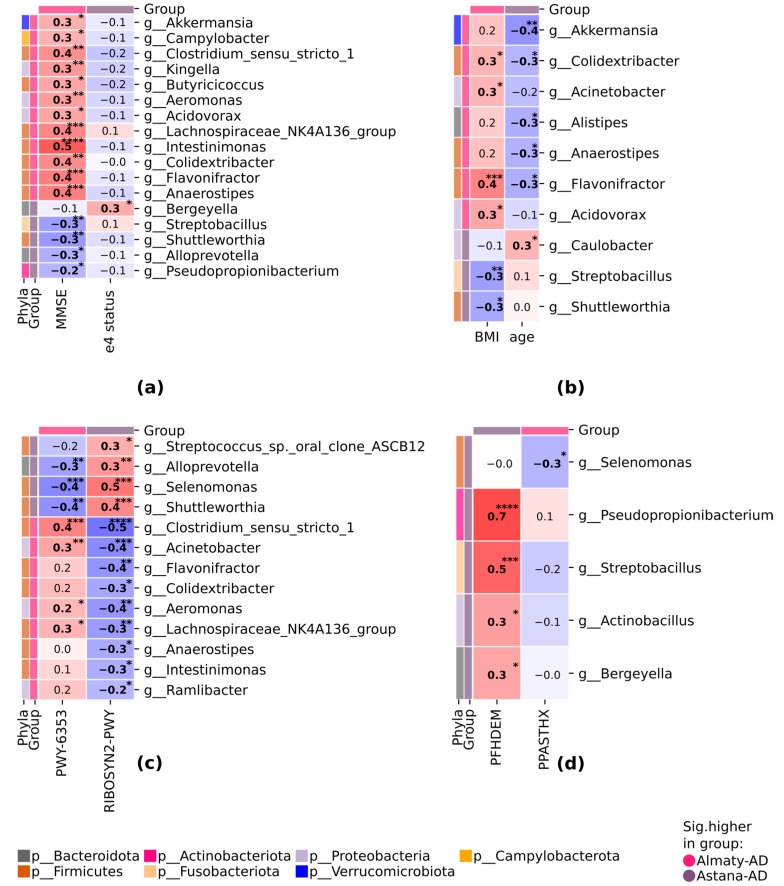
Significant correlations between differentially abundant features in AD subgroups in the Almaty and Astana regions: (**a**) between taxa, MMSE, and e4 status; (**b**) between taxa and demographic parameters; (**c**) between taxa and pathways; (**d**) between taxa, family history of dementia (PFHDEM), and self-reported depression (PPASTHX) status. Spearman’s ρ, *p* ≤ 0.05 for continuous parameters. Point-biserial r, *p* ≤ 0.05, for binary-continuous pairs; MMSE = Minimized Mental State Examination; BMI = body mass index. For differentially abundant features, a group with a higher relative abundance is highlighted: in coral, the relative abundance is higher in the Almaty-AD group, and in gray, it is higher in the Astana-AD group. * *p* ≤ 0.05, ** *p* ≤ 0.01, *** *p* ≤ 0.001, **** *p* ≤ 0.0001.

Correlation analysis within the main study group depending on regions revealed significant relationships at the genus level. The Almaty-AD subgroup demonstrated the most significant positive correlations between MMSE and taxa, such as *g__Lachnospiraceae_NK4A136_group*, *g__Intestinimonas*, *g__Colidextribacter*, *g__Flavonifractor*, and *g__Anaerostipes*. Taxon *g__Bergeyella* positively correlated with APOE_e4_status in the Astana-AD subgroup (Figure 5a).

Of note, relationships were identified between BMI, age, and certain taxa: *g_Flavonifractor*, *g_Colidextribacter* positively correlated with BMI and negatively correlated with age, while *g_Acinetobacter* and *g_Acidovorax* positively correlated with BMI. *g_Streptobacillus* and *g_Shuttleworthia* negatively correlated with BMI. *g_Akkermansia*, *g_Alistipes*, and *g_Anaerostipes* negatively correlated with age, while *g_Caulobacter* positively correlated with age (Figure 5b).

Relationships between taxa and metabolic pathways were disclosed, precisely, that two pathways, PWY-6353 and RIBOSYN2-PWY, demonstrated the most relationships with taxa at the genus level (Figure 5c). The remarkable trends were observed regarding *g__Clostridium_sensu_stricto_1*, *g__Acinetobacter*, *g__Aeromonas*, and *g__Lachnospiraceae_NK4A136_group* positively correlated with PWY-6353 in the Almaty-AD group, while *g__Alloprevotella*, *g__Selenomonas*, *g__Shuttleworthia* demonstrated opposite correlations in two study groups (Figure 5c).

PFHDEM (family history of dementia) positively correlated with *g_Pseudopropionibacterium*, *g_Streptobacillus*, *g_Actinobacillus*, and *g_Bergeyella*, while PPASTHX (self-reported depression) demonstrated a negative correlation with *g_Selenomonas* (Figure 5d).

The distribution of metabolic pathways in the Almaty-AD and Astana-AD subgroups is provided in Figure S3; only two pathways demonstrated statistical significance.

### 3.6. Feature Importance Analysis of AD and Control Groups

**Figure 6:**
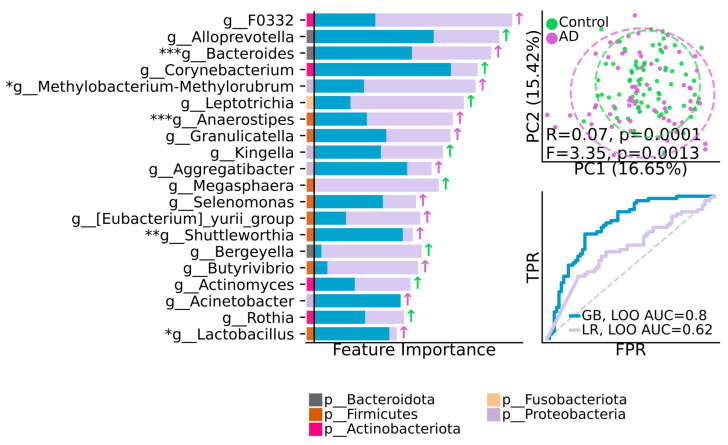
Feature importance analysis of AD and control groups. (**Left**): First 20 taxa, ranked by estimated importance for discriminating between groups. (**Upper right**): Principal coordinate analysis (PCoA) ordination based on the top 20 important taxa. Bray–Curtis dissimilarity with ANOSIM and PERMANOVA tests. (**Lower right**): AUC-ROC curve reflecting the degree of separability between groups during validation. GB = gradient boosting; LR = logistic regression; LOO = leave-one-out cross-validation; AUC = area under the curve. AUC > 0.5 suggests group separation, whereas AUC = 1 indicates absolute separation. Group with higher median abundance is indicated by (↑) in green for control and in purple for AD. * *p* ≤ 0.05, ** *p* ≤ 0.01, *** *p* ≤ 0.001.

The cross-validated feature importance analysis at all genera levels was performed to reveal the key taxa with the most predictable power among AD and control groups. Overall, twenty ranked by estimated importance between groups taxa demonstrated the key featured prediction with GB, LOO AUC = 0.8, LR, LOO AUC = 0.62 (Figure 6).

Five taxa, *g__Bacteroides* (*p* < 0.0001), *g__Methylobacterium-Methylorubrum* (*p* = 0.01), *g__Anaerostipes* (*p* < 0.0001), *g__Shuttleworthia* (*p* = 0.001), and *g__Lactobacillus* (*p* = 0.009), were statistically common in AD compared to the control group. g_F0332 demonstrated the leading prediction feature in the AD group, while *g_Alloprevotella* demonstrated the most predictable power in the control group. Notably, the majority of the key taxa belonged to the Firmicutes phylum (Figure 6).

## 4. Discussion

Despite numerous studies devoted to AD, a precise mechanism for the disease development still needs to be discovered. The scientific community currently targets the microbiome as a critical point in various diseases [34], including AD pathogenesis. This performed study provides the oral microbiome compartment peculiarities in Kazakhstanis in the interactions with clinical, demographic, and anamnestic data and laboratory parameters. The periodontal condition and its contribution to AD development and progression are still under investigation. The absence of significant differences in periodontitis between our study groups and any associations of periodontitis with AD corresponds to the recent review by Liccardo et al. about the debatable coexistence of these two diseases [35]. Particularly, our data did not reveal significant differences in the prevalence and abundance of known periodontal pathogens, the distribution of periodontal parameters, or the association of these parameters with the composition of the oral microbiome.

From the perspective of previous studies, our findings regarding oral microbiome composition in AD patients, precisely the bacterial richness, evenness, and dissimilarity, correspond to the Holmer et al. study [36]. Meanwhile, Wu et al. [37] and Yang et al. [38] reported results contradicting our identified lower oral microbiome diversity among AD patients. The recent studies of Fu et al. and Tadjoedin et al. observed no significant differences in alpha diversity and richness [39,40].

Relative abundance analysis revealed the downward trends in phyla *Proteobacteria*, *Actinobacteria*, and *Bacteroidota* in the AD group. At the same time, Firmicutes was significantly more abundant in AD patients, which aligns with the results of Chen et al. [41,42]. The results on the lower abundance of *s_Haemophilus_parainfluenzae*, *s_Prevotella_melaninogenica*, *s_Prevotella_histicola*, and *s__Actinomyces_oris* in AD patients are partly consistent with Liu et al. data [12]. However, the lower abandoned *Actinomyces* in this study were associated with the APOE_e4 genotype. Wu et al. observed the low abundance of this taxon in AD patients, corresponding to our findings [37]. In their study on Canadians, Cristea et al. also observed the *Actinomycetaceae* family’s lower abundance in AD [43]. *Actinomyces* is known as a pathogen associated with periodontitis development [44]. Our findings regarding *Prevotella’s* lower abundance, correspond to the Yang et al. study [38]. *Haemophilus_parainfluenzae* was recently associated with healthy oral conditions in the study of Moroccan adolescents with periodontitis, which is partly consistent with our data [45]. Noteworthy, our study results demonstrate a decrease in the level of periodontitis-associated bacteria in the AD group.

Our LefSe-based findings regarding *Lactobacillus* and *Lacticaseibacillus* contradict Wu et al.’s study [37], obtaining a lower abundance of these taxa. However, the recent animal study of Liu et al. demonstrated the protective feature of *Lactobacillus*; an increase of this taxon led to the attenuation of AD symptoms [46]. Interestingly, we did not observe the increase in *Porphyromonas gingivalis* and *Aggregatibacter actinomycetemcomitans* in AD patients despite several existing studies proving these taxa’s relation to AD disease, according to a recent review by Weber et al. [47] and Wan et al. [48]. However, our region-based analysis results revealed the increase of *Porphyromonas* and *Actynobaccillus* in the Astana-AD subgroup.

Our findings related to regional differences in AD patients’ oral microbiome compartment totally correspond to existing data on the variations of oral microbiome composition depending on geographical and environmental factors [49,50], even in one ethnic group [50]. However, in the absence of differences in species richness, nearly no differences were found from a functional side in our main group divided into regions. Consequently, we can assume that AD may have a peculiar oral microbiome composition.

Two pathways, HISTSYN-PWY (L-histidine biosynthesis) and PWY-2941 (L-lysine biosynthesis II), demonstrated opposite key correlations relating to AD development, a negative correlation with laboratory parameters such as cholesterol and LDL and a positive correlation with MMSE. Moreover, two bacteria classes, *Bacteroidia* and *Erysipelotrichia,* demonstrated the opposite correlations with mentioned pathways. Our finding corresponds to the Nielsen et al. recent metabolomic study, demonstrating that histidine metabolisms are downregulated in patients with AD [51]. Assuming that *Bacteroidia* may contribute to the downregulation of HISTSYN-PWY and that the latter is associated with the decrease in cholesterol level, which is known as a risk factor and the main culprit of AD [52,53,54], obtained results may be used in further new treatment target-searching studies. Existent data on the L-lysine biosynthesis II pathway reveal the twofold function of lysine in human health. On the one hand, it is known for its energy source and antioxidant capabilities [24]; on the other hand, according to Yuan et al., it enhances tumor growth [25].

Our featured important analysis results provided the specific taxa with the most predictable power regarding AD, according to a recent animal study by Xia et al. Bacteroides fragilis activating microglia leads to AD development [55]; *g__Methylobacterium-Methylorubrum*, according to Kovaleva et al., could cause the infectious process in individuals with a compromised immune system [56]; *g__Anaerostipes*, according to Tran et al., was associated with urine toxin production in chronic kidney disease [57]; *g__Shuttleworthia* was associated with periodontitis [58,59,60]; and *g__Lactobacillus*, according to a recent report by Rastogi and Singh, is known for its immune system-modulating trait [61]. Nevertheless, the ambivalent characteristic of this taxon regarding autoimmune and other chronic disease development should not be underestimated [62].

Our findings highlight future research directions in the field of AD disease and the association of oral microbiome with its development, replenishing the data on AD’s new potential biomarkers and treatment approaches projects from the point of oral microbiome stamp and metabolic pathways. The study of possible mechanisms linking the oral microbiota and AD incidence will advance the understanding of its pathology and may serve as a basis for the development of prevention strategies associated with oral health. Certainly, there are several limitations in our study: first, we performed a case-control study, and further longitudinal research with a larger AD cohort is needed to provide higher reliability of the obtained data; secondly, the sequence analysis was performed using 16S rRNA, and further Shotgun sequencing is required to gain more accurate data.

## 5. Conclusions

By our reckoning, the presented study is the first study focused on oral microbiome compartments in AD patients performed in Kazakhstan and Central Asia. Our findings demonstrate the decrease in periodontitis-associated bacteria in the AD group, along with the absence of periodontitis and AD relationships in our cohort. However, certain taxa and metabolic pathways demonstrated a promising perspective with regard to searching for new biomarkers, which requires further studies to affirm and fortify the obtained results.

## Figures and Tables

**Table 1:** Responders’ clinical and demographic characteristics.

Parameters	AD (*n* = 64)	Control (*n* = 71)	*p*-Value
Age (Me, years, Q1–Q3)	68 (63–74)	67 (61–72)	*p* = 0.113
Race (n, %)			*p* = 0.913
Kazakh	48 (75%)	52 (73.2%)	
Non-Kazakh	16 (25%)	19 (26.8%)	
Gender (n, %)			*p* = 1.000
Male (n, %)	16 (25%)	17 (23.9)	
Female (n, %)	48 (75%)	54 (76.1)	
BMI (Me, kg/m^2^, Q1–Q3)	24.7 (21.36–28.7)	26.3 (23.04–30.48)	*p* = 0.187
Periodontitis (n, %)	56 (87.5%)	58 (81.7%)	*p* = 0.476
History of smoking (n, %)	13 (20.3%)	10 (14.1%)	*p* = 0.337
APOE4 carrier (n, %)	26 (41.3%)	19 (27.1%)	*p* = 0.124
MMSE (mini-mental state examination), (Me, score, Q1–Q3)	21 (13–22)	29 (29,30)	*p* < 0.0001 *
Fasting glucose (mmol/L)	5.07 (4.62–5.47)	5.18 (4.76–5.86)	*p* = 0.135
TC (mmol/L)	4.84 (4.20–5.15)	5.53 (4.48–6.10)	*p* = 0.001 *
TG (mmol/L)	1.15 (0.84–1.55)	1.48 (0.93–2.09)	*p* = 0.01 *
LDL (mmol/L)	2.79 (2.37–3.43)	3.31 (2.77–3.86)	*p* = 0.004 *
ALT (IU/L)	11.85 (7.9–17.2)	16.65 (10.6–22.6)	*p* = 0.004 *
AST (IU/L)	17.55 (15.3–20.6)	18.55 (16.7–22.6)	*p* = 0.052 *
CRP (mg/L)	1.7 (0.6–3.55)	1.45 (0.7–3.9)	*p* = 0.857
PFHDEM (family history of dementia)	2 (3.1%)	3 (4.3%)	*p* = 1.000
PPASTHX (self-reported depression)	26 (40.6%)	23 (32.4%)	*p* = 0.581
PTOLDBP (self-reported blood pressure)	32 (50%)	38 (53.5%)	*p* = 0.365
PTOLDHRT (self-reported heart disease)	14 (21.8%)	16 (22.5%)	*p* = 0.874
PCVA (self-reported stroke), (n (%))	5 (7.8%)	4 (5.6%)	*p* = 1.000
PTIA (self-reported TIA (transient ischemic attack))	5 (7.8%)	9 (12.7%)	*p* = 0.268
PLOC (self-reported brain injury)	11 (17.2%)	11 (15.5%)	*p* = 0.97
PTOLDDM (self-reported diabetes)	7 (10.9%)	7 (9.8%)	*p* = 1.000

Note: * *p* ≤ 0.05.

**Table 2:** AD region-based subgroups’ clinical and demographic characteristics.

Parameters	Almaty-AD (*n* = 34)	Astana-AD (*n* = 30)	*p*-Value
Age (Me, years, Q1–Q3)	66 (62–72)	71 (67–80)	*p* = 0.002 *
Race (n, %)			*p* = 0.679
Kazakh	24 (70.6%)	24 (80.0%)	
Non-Kazakh	10 (29.4%)	6 (20.0%)	
Gender (n, %)			*p* = 0.247
Male (n, %)	11 (32.4%)	5 (16.7%)	
Female (n, %)	23 (67.6%)	25 (83.3%)	
BMI (Me, kg/m^2^, Q1–Q3)	26.9 (23.4–29.0)	23.4 (21.1–27.9)	*p* = 0.03 *
Periodontitis (n, %)	29 (85.3%)	27 (90.0%)	*p* = 0.712
History of smoking (n, %)	10 (29.4%)	3 (10%)	*p* = 0.054
APOE_e4_carrier (n, %)	10 (29.4%)	16 (53.3%)	*p* = 0.06
MMSE (mini-mental state examination), (Me, score, Q1–Q3)	22 (20–24)	14.5 (4–21)	*p* = 0.0002 *
Fasting glucose (mmol/L)	5.11 (4.89–5.62)	4.76 (4.4–5.33)	*p* = 0.041 *
TC (mmol/L)	4.92 (4.44–5.15)	4.64 (3.97–4.99)	*p* = 0.100
TG (mmol/L)	1.16 (0.81–1.55)	1.09 (0.84–1.52)	*p* = 0.568
LDL (mmol/L)	2.7 (2.41–3.08)	3.04 (2.14–3.46)	*p* = 0.938
ALT (IU/L)	12 (7.1–16.4)	11.75 (8.4–17.5)	*p* = 0.438
AST (IU/L)	17.75 (16.1–20.6)	16.65 (13.8–20.0)	*p* = 0.324
CRP (mg/L)	1.55 (0.47–2.8)	2.15 (0.62–5.03)	*p* = 0.159
PFHDEM (family history of dementia)	0 (0%)	2 (6.7%)	*p* = 0.05 *
PPASTHX (self-reported depression)	21 (61.8%)	5 (16.6%)	*p* = 0.0006 *
PTOLDBP (self-reported blood pressure)	19 (55.9%)	13 (43.3%)	*p* = 0.627
PTOLDHRT (self-reported heart disease)	7 (20.6%)	7 (23.3%)	*p* = 0.759
PCVA (self-reported stroke), (n (%))	2 (5.9%)	3 (10.0%)	*p* = 0.643
PTIA (self-reported TIA (transient ischemic attack))	3 (8.8%)	2 (6.7%)	*p* = 1.000
PLOC (self-reported brain injury)	8 (23.5%)	3 (10.0%)	*p* = 0.497
PTOLDDM (self-reported diabetes)	3 (8.8%)	4 (13.3%)	*p* = 0.454

Note: * *p* ≤ 0.05.

## Data Availability

Sequence data of the current study have been deposited in the NCBI BioProject; the primary accession number is PRJNA1062161.

## Supplementary Materials

The following supporting information can be downloaded at https://www.mdpi.com/article/10.3390/pathogens13030195/s1: Figure S1. Distribution of metabolic pathways in AD and control groups; Figure S2. Distribution of metabolic pathways in the Almaty-AD and Astana-AD subgroups; Figure S3. Differentially abundant pathways in AD subgroups in Almaty and Astana regions. Relative abundance of differentially abundant pathways and 95% confidence interval (CI) for the difference between their medians. Mann-Whitney U test and CI computed using the Hodges-Lehmann estimator. Only differences in pathways with non-overlapping CIs are considered significant. * *p* ≤ 0.05; Table S1. Periodontal parameters in AD and control groups; Table S2. Periodontal parameters by gender in AD and control groups; Table S3. Correlations between significantly abundant taxa and metabolic pathways.
