# Supplementary material for: Oral Microbiome Stamp in Alzheimer’s Disease
Source: Pathogens. 2024 Feb 23;13(3):195. doi: 10.3390/pathogens13030195 (PMC10975384; doi:10.3390/pathogens13030195)
Supplement: Supplementary file 1 [file pathogens-13-00195-s001.zip › Table S1.pdf]

Table S1. Periodontal parameters in AD and control groups.

Parameters	AD∣Periodontitis (*n* = 56)	Control∣Periodontitis (*n* = 58)	*p*-value
**Bone loss (%)**	73 (59–79)	72.5 (60.25–76.0)	$p = 0.691$
**BoP (score) 1/2/3**	32/20/4 (57.1%, 35.7%, 7.1%)	31/21/6 (53.4%, 36.2%, 10.3%)	$p = 0.817$
**PPD (mm)**	7 (6-7)	7 (6–7)	$p = 0.386$
**CAL (mm)**	3.5 (3-4)	4(3–4)	$p = 0.817$
**GR (mm)**	−3 (-4–−2.5)	3 (−3.5–−2)	$p = 0.454$
**Number of remaining teeth**	9.5 (8–16)	10.5 (9–15)	$p = 0.580$
