# Supplementary material for: Oral Microbiome Stamp in Alzheimer’s Disease
Source: Pathogens. 2024 Feb 23;13(3):195. doi: 10.3390/pathogens13030195 (PMC10975384; doi:10.3390/pathogens13030195)
Supplement: Supplementary file 1 [file pathogens-13-00195-s001.zip › Table S2.pdf]

Table S2. Periodontal parameters by gender in AD and control groups.

Parameters	Female (*n* = 102)	Male (*n* = 33)	*p*-value
**Periodontitis**	88 (86%)	26 (78%)	$p = 0.406$
	**Female∣Control (*n* = 54)**	**Male∣Control (*n* = 17)**	***p*-value**
**Periodontitis in Control group**	45 (83.3%)	13 (76.4%)	$p = 0.495$
	**Female∣AD (*n* = 48)**	**Male∣AD (*n* = 16)**	***p*-value**
**Periodontitis in AD group**	43 (89.5%)	13 (81.2%)	$p = 0.401$
