# Supplementary figures and images for: Oral Microbiome Stamp in Alzheimer’s Disease
Source: Pathogens. 2024 Feb 23;13(3):195. doi: 10.3390/pathogens13030195 (PMC10975384; doi:10.3390/pathogens13030195)

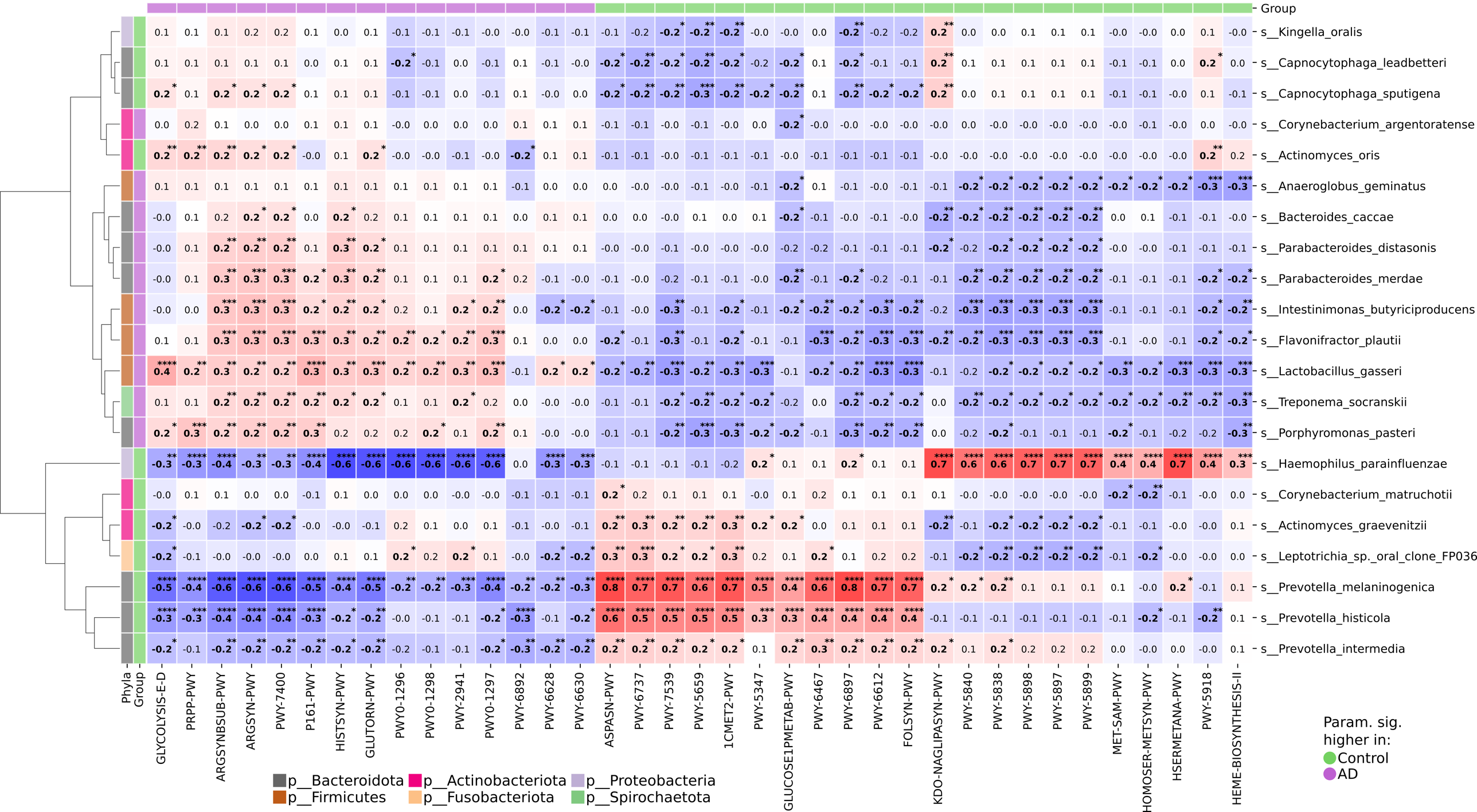

Supplement: Supplementary file 1 [file pathogens-13-00195-s001.zip › Figure S1.png]

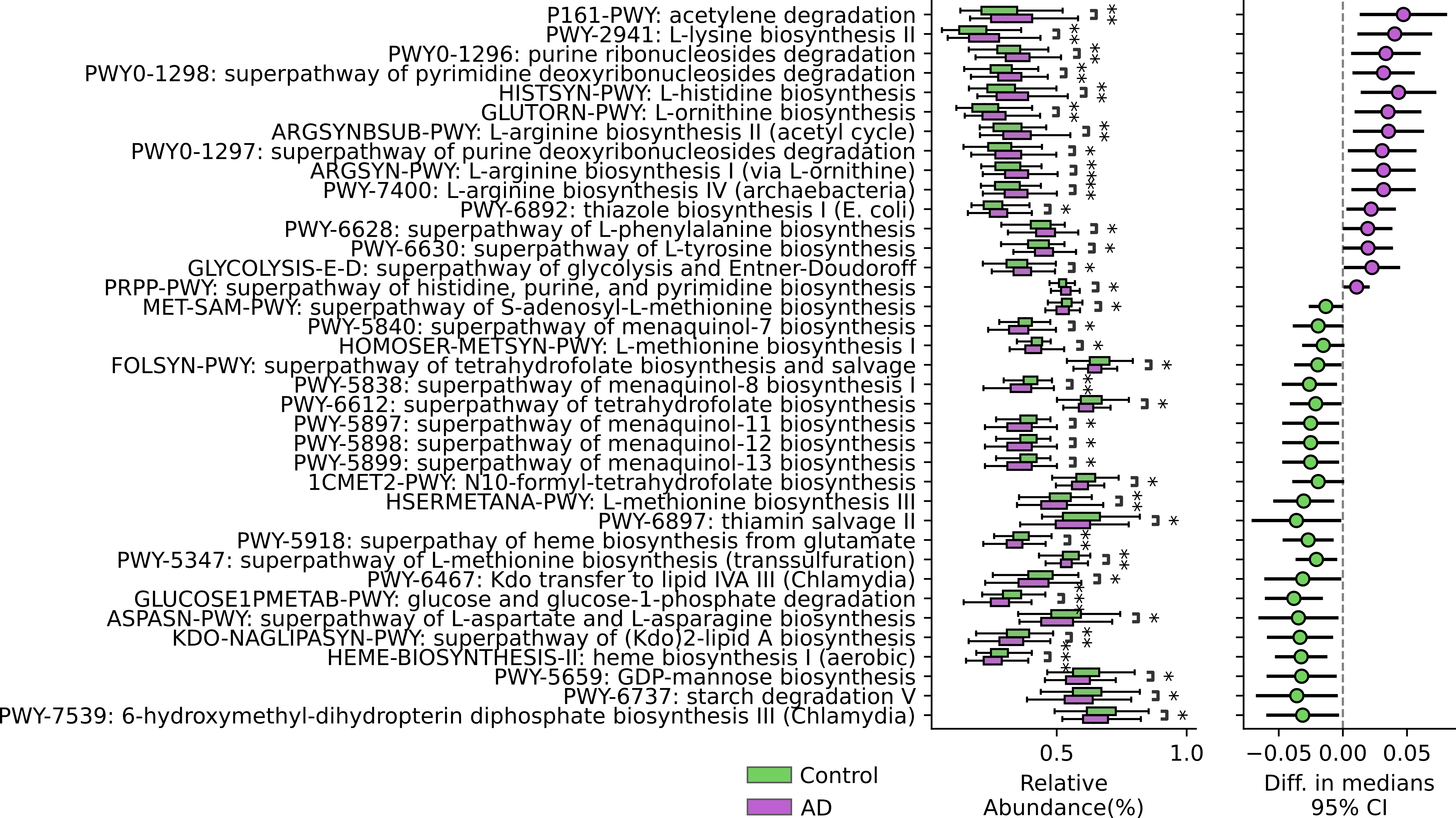

Supplement: Supplementary file 1 [file pathogens-13-00195-s001.zip › Figure S2.png]

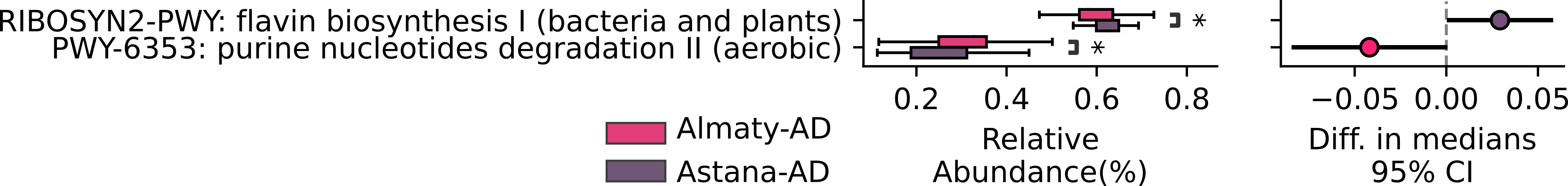

Supplement: Supplementary file 1 [file pathogens-13-00195-s001.zip › Figure S3.png]
